# How Is Cultural Intelligence Related to Human Behavior?

**DOI:** 10.3390/jintelligence10010003

**Published:** 2022-01-07

**Authors:** Moh. Alifuddin, Widodo Widodo

**Affiliations:** 1 Informatics Engineering Department, STMIK Handayani Makassar, Makassar 90231, Indonesia; jurnalalifuddin@gmail.com; 2 Social Science Education Department, Postgraduate Faculty, Universitas Indraprasta PGRI, Jakarta 12530, Indonesia

**Keywords:** cultural intelligence, interpersonal communication, psychological capital, organizational citizenship behavior

## Abstract

Cultural intelligence is an individual’s ability to recognize, understand, and adapt to cross-cultural contexts in order to change his or her self-capacity. Hence, this study explores the relationship between cultural intelligence and interpersonal communication, psychological capital (PsyCap), and organizational citizenship behavior (OCB) among teachers in Indonesia and investigates the possibility of finding relevant new models. A Likert questionnaire was used to collect research data. The research participants included 450 Indonesian junior high school teachers selected by accidental sampling. Structural equation modeling (SEM) was used for data analysis, supported by descriptive statistics and correlational matrices. The results indicate that cultural intelligence is significantly related to teachers’ interpersonal communication, PsyCap, and OCB. Additionally, this study also produces a new model regarding the relationship between cultural intelligence and a teacher’s OCB, mediated by interpersonal communication and PsyCap. Therefore, researchers and practitioners can discuss and adopt a new empirical model to increase cultural intelligence.

## 1. Introduction

The benefits of intelligence for life should not be doubted. Various studies have shown that intelligence contributes to individuals’ livelihood and has implications for groups and organizations. One of them is cultural intelligence. Scholars have claimed that cultural intelligence is a measure of intercultural competence (Ang and Van Dyne 2008; Matsumoto and Hwang 2013; Leung et al. 2014; Yari et al. 2020), including work-related outcomes, such as job satisfaction, work adjustment, job performance (Schlaegel et al. 2021; Zhao et al. 2020; Akhal and Liu 2019; Baluku et al. 2019; Rockstuhl and Van Dyne 2018; Henderson et al. 2018), and knowledge sharing (Stoermer et al. 2021). The concept of cultural intelligence is based on the idea that acting intelligently within diverse cultures may require more than general intelligence and its subfactors (Ang et al. 2020). Conceptually, cultural intelligence is an individual’s capability to function effectively in culturally diverse contexts (Earley and Ang 2003; Ang and Van Dyne 2008). This definition of cultural intelligence—as a capability—emphasizes a person’s potential to be effective across a wide range of intercultural contexts (Ang et al. 2015). Yari et al. (2020) define cultural intelligence as the ability to succeed in complex cross-cultural environments through knowledge or cognition, motivation, and behaviors. Cultural intelligence also refers to being skilled and flexible regarding the understanding of a culture, learning more about it from ongoing interactions, and gradually reshaping one’s thoughts to be more sympathetic to the culture and behaviors of others, so as to be more skilled and appropriate when interacting with them (Thomas and Inkson 2017). Cultural intelligence also reflects an individual’s ability to help in recognizing the specificities of many cultures, and to understand and adapt to cross-cultural contexts (Berraies 2020). Sternberg et al. (2021) describe cultural intelligence as one’s ability to adapt when confronted with problems arising in interactions with people of diverse cultures. However, cultural intelligence also draws upon abstract analytical abilities, as one must analyze situations that, unlike many practical problems, are rather removed from one’s everyday experience. Cultural intelligence draws on creative abilities since the problems one confronts are more novel than one would confront in typical tests as well as life situations. Cultural intelligence includes the ability of individuals and organizations to adapt (Solomon and Steyn 2017) to an external environment consisting of various cultures. Therefore, cultural intelligence has a strategic position in social relations involving many people from various cultural backgrounds. For example, in learning activities, teachers’ cultural intelligence is required to understand the condition of students from various cultural backgrounds and then adapt and position themselves appropriately among students. Understanding, adapting, and positioning oneself is important, since culture has been shown to influence one’s perception and cognition (Kastanakis and Voyer 2014). Moreover, human behavior is strongly influenced by a content-related bias favoring culture (Cronk 2017). In fact, people tend to behave preferentially towards people with the same cultural background. Although this is not an error from a specific cultural perspective, it can hinder social relations with people from different cultural backgrounds. Overall, this tendency can disrupt cooperation, collaboration, and partnership, and stimulate interpersonal or intergroup conflicts. Hence, an understanding of various cultures is an essential skill for a person to increase his or her capacity, including interpersonal communication, PsyCap, and OCB, in order to adapt when interacting with people from different cultural backgrounds. Furthermore, this skill is beneficial for achieving life goals with a greater chance of success. Based on these issues, this research focuses on efforts to reveal the influence of cultural intelligence on interpersonal communication, PsyCap, and OCB, which are much needed by teachers in their teaching tasks. Furthermore, this study investigates the relationship between interpersonal communication and PsyCap with OCB, to find novel and relevant models regarding the relationship between cultural intelligence and OCB, mediated by interpersonal communication and PsyCap. This is crucial for teachers in Indonesia, who have quite diverse cultural backgrounds. Specifically, Indonesia has 1340 ethnic groups, each of which has its own unique culture. The Javanese are the largest ethnic group making up 41% of the population. Thus, teachers in Indonesia have diverse cultural backgrounds. Some teachers teach in schools with the same cultural base in their respective regions, while others, in different regions, teach in schools with students from different cultures as well as students with the same or different cultural backgrounds. In Indonesia, many teachers study at universities in other regions with different cultural backgrounds. This setting causes teachers to be involved and become part of the acculturation and cultural assimilation process that can impact their perspective on culture as the core of cultural intelligence. Ang et al. (2020) mention the four-factor model of cultural intelligence: metacognitive, cognitive, motivational, and behavioral. (1) Metacognitive consists of planning, awareness, and checking; (2) cognitive consists of culture-general knowledge and context-specific knowledge; (3) motivational consists of intrinsic interest, extrinsic interest, and self-efficacy to adjust; and (4) behavioral consists of verbal behavior, nonverbal behavior, and speech acts. Meanwhile, Thomas and Inkson (2017) proposed three indicators of cultural intelligence: knowledge, mindfulness, and behavior skills. Knowledge refers to individuals’ knowledge about cultures, including what culture is, how cultures are different, and how culture influences behavior skills. Mindfulness is related to being open-minded and using the context of the situation to support understanding. Behavior skills relate to demonstrating appropriate behaviors or social skills in new cultural settings. Researchers use the four-factor model from Ang et al. (2020) widely as an indicator for measuring cultural intelligence constructs. However, this study used three indicators: knowledge, mindfulness, and behavior skills, from Thomas and Inkson (2017), with the rationale that they are more suitable for Indonesian teachers, who have very diverse cultural backgrounds.

### 1.1. Cultural Intelligence and Interpersonal Communication

Cultural intelligence, among other things, is related to interpersonal communication. Investigations across various fields have found that cultural intelligence affects interpersonal communication (Mukherji et al. 2016; Ahmadian and Amirpour 2018; Bahrami and Narafshan 2018; Henderson et al. 2018; Bostan et al. 2021). Importantly, interpersonal communication is essential for individual activities. For teachers, interpersonal communication should help develop social relationships among all school members, especially for building dynamic interactions with students. Teachers with interpersonal communication skills can easily establish interpersonal relationships with students so that interactions between teachers and students can take place during the learning process. Interpersonal communication can influence injunctive norms, self-efficacy, behavioral intentions (Duong et al. 2021), and job performance (Muhammad et al. 2018; Saraih et al. 2019). Furthermore, interpersonal communication also affects organizational effectiveness (Mukhtar and Prasetyo 2020). Interpersonal communication is a flow or exchange of information between individuals in face-to-face and group settings (Gibson et al. 2012). Interpersonal communication also refers to the pattern flow of communication, relationships, and understandings developed over time among people, rather than focusing on the individual and whether a specific message is received as intended by the sender. This pattern involves the ongoing flow of verbal, written, and nonverbal messages between two people or between one person and others (Hellriegel and Slocum 2011). In reality, interpersonal communication can take many formal and informal channels through numerous media and technologies (Hitt et al. 2011). DeVito (2016) mentions five indicators of interpersonal communication: openness, empathy, supportiveness, positiveness, and equality. Openness refers to the willingness to express oneself and act honestly with other people’s messages. Empathy reflects what other people feel—experiencing what another person is experiencing from his or her point of view without losing one’s identity. Supportiveness refers to having an uncertain and open-minded attitude and being willing to listen to opposing points of view, to change one’s position, and to assist in creating a supportive environment. Positiveness refers to possessing a positive attitude and praising interaction partners. Equality is related to the view that disagreement is seen as an easier way to solve problems. These indicators can be well developed if the teacher has cultural intelligence, which is reflected in his or her knowledge, mindfulness, and behavior skills within the context of culture. For instance, teachers who have knowledge about cultures, such as what culture is, how cultures are different, and how culture influences behavior and skills, will tend to be open and empathic and uphold equality principles in fostering communication with other people (including students) from various cultural backgrounds. Meanwhile, teachers with a good mindfulness—reflected in being open-minded and using the context of situations to support their understanding—tend to be supportive and positive towards other people in their interpersonal communication patterns. Therefore, the following hypothesis (H) can be formulated:

**Hypothesis** **(H1).** *Cultural intelligence has a relationship with the teacher’s interpersonal communication*.

### 1.2. Cultural Intelligence and PsyCap

Cultural intelligence is also related to PsyCap. A previous study showed that cultural intelligence significantly affects PsyCap (Chen and Chen 2018; Imran and Shahnawaz 2020; Jiony et al. 2021). This indicates that cultural intelligence, reflected in knowledge, mindfulness, and behavior skills regarding culture, can enhance PsyCap. PsyCap is a basic competency that plays a vital role in employees’ handling of awareness and achieving efficiency at work (Cavus and Gokcen 2015). According to Goertzen and Whitaker (2015), PsyCap offers a framework to understand human assets that can be useful in actualizing the human potential. Luthans and Youssef-Morgan (2017) state that PsyCap goes beyond the human capital theory, that answers the question: “what do we know?”, and the social capital theory: “who do we know?” It also answers the ultimate questions that all individuals pose themselves: “who are we?” PsyCap refers to an individual’s positive and developmental state characterized by self-efficacy, optimism, hope, and resilience (Luthans and Youssef 2004; Luthans and Youssef-Morgan 2017). Self-efficacy refers to individuals’ belief in their capabilities to complete tasks. Optimism is based on the clear appraisal and judgment of current situations and understanding what can be done in such situations. Hope is the individuals’ belief in their determination to achieve their goals and find possible pathways to overcome difficulties. Resilience refers to the ability to recover from adversity or setbacks, proactively rising to challenges, and adapting to an ever-changing business environment (Tang et al. 2019). Several studies across various fields and contexts have indicated that Psycap contributes to an individual’s life and career. In the academic field, Carmona-Halty et al. (2019) reported that Psycap determines academic performance. Recent studies by Imran and Shahnawaz (2020) also revealed that PsyCap influences performance. Moreover, recent studies also claim that PsyCap is significantly related to career outcomes (Baluku et al. 2021) and innovative behavior (Slatten et al. 2020; Wang et al. 2021). These previous studies confirm that PsyCap is significantly important for individuals and organizations, especially teachers in a school organization context. Hence, it is crucial that this topic is researched and extensively discussed, in order to explore and analyze the factors that influence it, including cultural intelligence. The contextualization of the relationship between cultural intelligence and PsyCap can be viewed in the context of teachers with high knowledge and mindfulness regarding cultures to be self-efficacious, optimistic, and resilient. For example, a teacher with high knowledge of culture(s), including what culture is, how cultures are different, and how culture influences behavior and skills, will have high self-efficacy, which will be reflected in their belief in their capabilities to complete tasks. Likewise, teachers with high mindfulness—marked by being open-minded and using situational contexts to support their understanding—tend to have increased optimism (manifested in the clear appraisal and judgment of current situations and an understanding of what can be done in such situations) and high resilience to recover from adversity or setbacks, and can proactively rise to challenges and adapt to ever-changing environments. Therefore, the following hypothesis can be formulated:

**Hypothesis** **(H2).** *Cultural intelligence has a relationship with a teacher’s PsyCap*.

### 1.3. Cultural Intelligence and OCB

Several previous studies have also revealed that cultural intelligence is not only related to interpersonal communication and PsyCap but also to OCB. For instance, Narayanan and Nirmala (2016) and Shafieihassanabadi and Pourrashidi (2019) claimed that cultural intelligence affects OCB. Other studies by Mehdipour et al. (2019) and Kadam et al. (2021) also indicated that cultural intelligence had a positive impact on OCB. However, an opposite study investigating the relationship between OCB and cultural intelligence conducted by Popescu et al. (2018) indicated the existence of some influencing OCB components on cultural intelligence. The intensity of the influence was weak, and so OCB cannot be considered a cultural intelligence predictor. This shows that cultural intelligence is an essential antecedent for OCB; therefore, cultural intelligence dimensions, such as knowledge, mindfulness, and behavior skills regarding culture, can impact OCB. According to Cascio (2016), OCB refers to discretionary behaviors performed outside of one’s formal role that can help other employees perform their job or show support and conscientiousness towards an organization. OCB consists of employee behavior that goes beyond the call of duty and exceeds formal job duties, such as cooperation and helpfulness to others that supports the organization’s social and psychological context; however, it is often necessary for organizational survival (Kreitner and Kinicki 2013; McShane and Von Glinow 2020). Meanwhile, Schultz and Schultz (2016) described OCB as putting forth effort, i.e., doing more for your employer than the minimum job requirements, including taking on additional assignments, voluntarily assisting others at work, being up to date with the developments in one’s field or profession, following company rules (even when no one is looking), promoting and protecting the organization, and maintaining a positive attitude and tolerating work inconveniences. Organ et al. (2006) mention OCB’s indicators: altruism, conscientiousness, sportsmanship, courtesy, and civic virtue. Altruism is related to helping others who may be having difficulties related to organizational or personal tasks. Conscientiousness refers to an effort to exceed the organization’s expectations. Sportsmanship is about tolerating conditions that are less than ideal in the organization. Courtesy is about maintaining good relations with others to avoid interpersonal problems. Finally, civic virtue refers to being responsible for organizational life. The relationship between cultural intelligence and OCB, for example, can be judged from the way in which mindfulness as a cultural intelligence indicator is reflected in one’s open-mindedness and use of a situational context to support understanding, which can potentially stimulate some OCB indicators, such as conscientiousness, courtesy, and civic virtue. Furthermore, behavior skills—the ability to demonstrate appropriate behaviors or social skills in new cultural settings—also contribute to increasing altruism and sportsmanship in an OCB context. Recently, OCB has received greater attention among academics, researchers, and practitioners, since it is vital for individuals, e.g., teachers in a school organization context. Several studies have indicated the power of OCB. Widodo and Gustari (2020), for instance, showed that OCB influences teachers’ innovative behavior in an educational context. Other studies have indicated that OCB affects employees’ productivity (Barsulai et al. 2019), performance (Hidayah and Harnoto 2018), and organizational performance (Aval et al. 2017). This means that OCB is essential for teachers; therefore, it requires urgent investigation, primarily related to cultural intelligence. Based on the argument and above-mentioned studies, the following hypothesis can be formulated:

**Hypothesis** **(H3).** *Cultural intelligence has a relationship with a teacher’s OCB.*

### 1.4. The Relationship between Interpersonal Communication and PsyCap with OCB

Several previous studies—throughout many countries across various fields and contexts—have shown that interpersonal communication, apart from being influenced by cultural intelligence, also affects OCB. For example, studies conducted by Ezerman and Sintaasih (2018), Putra (2018), Herfina and Wulandari (2019), Nofia et al. (2019), and Syamsudin and Retnowati (2019) claimed that interpersonal communication is related to OCB. This indicates that adequate interpersonal communication skills reflected in openness, empathy, supportiveness, positiveness, and equality (DeVito 2016) can lead to a high OCB, which is manifested as altruism, conscientiousness, sportsmanship, courtesy, and civic virtue (Organ et al. 2006). For example, teachers with high empathy, reflected by their sense of what other people feel, and experiencing what others are experiencing from their perspective (without losing one’s identity), will tend to have high altruism, which is reflected in their willingness to help others facing difficulties related to organizational and personal tasks. Likewise, teachers who have strong supportiveness—that is, who have an uncertain and open-minded attitude and who are willing to listen to opposing points of view, change their position, and assist in creating a supportive environment—can support their sportsmanship. This provides tolerance in less-than-ideal conditions within an organization. Accordingly, interpersonal communication indicators can influence OCB indicators.

In addition, other studies have reported that PsyCap, apart from being influenced by cultural intelligence, can also affect OCB (e.g., Kong et al. 2018; Aderibigbe and Mjoli 2018; El-Zohiry and Abd-Elbaqy 2019; Nawaz and Abid 2019; Adillah et al. 2019; Yildiz 2019; Chamisa et al. 2020; Rodríguez-Cifuentes et al. 2020; Da et al. 2021). This confirms that an adequate PsyCap, reflected as self-efficacy, optimism, hope, and resilience, can stimulate OCB, consisting of altruism, conscientiousness, sportsmanship, courtesy, and civic virtue (Organ et al. 2006). For example, teachers with high self-efficacy—reflected, for example, in a strong belief in their capacity to complete school tasks—tend to possess high conscientiousness, that can exceed a school’s expectations. Likewise, teachers with high hope, marked by a strong belief in their determination to achieve their goals and find possible pathways to overcome difficulties, also tend to have high civic virtue in the form of high responsibility for organizational life. Based on these studies and the above-mentioned arguments, the following hypothesis can be formulated:

**Hypothesis** **(H4).** *Interpersonal communication has a relationship with a teacher’s OCB*.

**Hypothesis** **(H5).** *PsyCap has a relationship with a teacher’s OCB*.

## 2. The Current Study

The current study focuses on investigating the relationship between cultural intelligence and human behavior—specifically, interpersonal communication, PsyCap, and OCB. Moreover, it focuses on the relationship between interpersonal communication and PsyCap on OCB and seeks novel and relevant models related to mediating the role of interpersonal communication and PsyCap on the relationship between cultural intelligence and OCB. To achieve this goal, using SEM analysis and research participants among teachers in Indonesia, we seek to confirm results from previous studies, which are used as the basis for building the research hypotheses of this study—namely the influence of cultural intelligence on interpersonal communication (Bostan et al. 2021), PsyCap (Chen and Chen 2018), and OCB (Shafieihassanabadi and Pourrashidi 2019). Then, we uncover the influence of interpersonal communication on OCB (Nofia et al. 2019; Syamsudin and Retnowati 2019) and the influence of PsyCap on OCB (Chamisa et al. 2020; Rodríguez-Cifuentes et al. 2020; Da et al. 2021). Finally, we hope to find the relationship between cultural intelligence and OCB, mediated by interpersonal communication and PsyCap, in order to build novel and relevant models from our research results.

## 3. Materials and Methods

### 3.1. Participants

The research participants (sample) consisted of 450 junior high-school teachers spread across eight provinces in Indonesia, namely Jakarta, Banten, West Java, Central Java, Riau Islands, Lampung, East Kalimantan, and East Nusa Tenggara. Moreover, the teachers were from 34 provinces in Indonesia, representing at least eight main ethnic groups—Betawi, Javanese, Sundanese, Bedouin, Kutai, Malay, Lampung, and Alor—forming the basis of cultural intelligence. From each province, four schools (two public schools and two private schools) were taken from four different districts; therefore, a total of 32 schools were included in this study. In total, 825 teachers from the 32 schools were used as the sample frame (population). Of these, 450 teachers voluntarily filled out the complete questionnaire during the research (Widodo 2019) and naturally became research participants. Their profiles are presented in Table 1. The majority of participants were female (68.67%), aged 26–35 years (35.33%), with a bachelor’s degree (90%), married (80%), and with teaching experience ≥16 years (31.11%).

### 3.2. Procedure and Materials

This research uses a quantitative approach with a survey method. Using a Likert scale, a questionnaire was employed to collect data with five options: strongly disagree, disagree, neutral, agree, and strongly agree. Since this research took place during the COVID-19 pandemic—which required all participants and researchers to comply with health protocols, especially social distancing—the survey was conducted online using Google Forms, which can be shared via the WhatsApp application (on the teacher group WhatsApp network). The questionnaire was constructed by researchers based on the theoretical dimensions or indicators of the experts. The cultural intelligence indicators were: knowledge (Know), mindfulness (Mind), behavior skill (BS) (Thomas and Inkson 2017); for interpersonal communication: openness (Open), empathy (Emp), supportiveness (Sup), positiveness (Pos), and equality (Equ) (DeVito 2016); for Psycap: self-efficacy (S-E), optimism (Opt), hope (Hop), and resilience (Res) (Luthans and Youssef-Morgan 2017); for OCB: altruism (Alt), conscientiousness (Con), sportsmanship (Spo), courtesy (cour), and civic virtue (CV) (Organ et al. 2006). Cultural intelligence consists of six items with a corrected item-total correlation coefficient between .445 and .798 and an alpha coefficient of .828. Interpersonal communication consists of eight items with a corrected item-total correlation coefficient between .458 and .712 and an alpha coefficient of .830. PsyCap consists of 12 items with a corrected item-total correlation coefficient between .530 and .869 and an alpha coefficient of .920. OCB consists of 10 items with a corrected item-total correlation coefficient between .497 and .765 and an alpha coefficient of .911. All items have corrected item-total correlation coefficients of >.361, and all variables have an alpha coefficient of >.70; therefore, it is valid and reliable as a research instrument (Van Griethuijsen et al. 2015; Hair et al. 2018).

### 3.3. Data Analysis

The data analysis was conducted by structural equation modeling (SEM) supported by correlational and descriptive statistics. The significance of the path coefficient of direct correlation was tested using the Student’s *t*-test, while the Sobel test (Z) was used for the path coefficient of indirect correlation (Abu-Bader and Jones 2021). Descriptive and correlational analyses were performed by SPSS version 26, while SEM analysis was performed by LISREL 8.80.

## 4. Result

The descriptive statistical analysis result for the four research variables indicates the mean value indicators of cultural intelligence from the lowest to the highest, in succession: BS = 8.46, Know = 8.85, and Mind = 9.04; interpersonal communications: Pos = 4.18, Equ = 4.23, Emp = 8.40, Sup = 8.72, and Open = 9.09; PsyCap: Hop = 11.09, Opt = 11.74, Sel = 12.56, and Res = 13.02; OCB: Sport = 7.82, Cons = 7.97, Alt = 7.98, CV = 7.99, and Court = 8.90. The standard deviation (std. dev) values of the cultural intelligence indicators (from lowest to highest, in succession) are: Mind = .925, BS = 1.107, and Know = 1.127; interpersonal communication = Pos= 7.33, Equ = 7.44, Open = 1.020, Sup = 1.140, and Emp = 1.261; PsyCap: Res = 1.383, Sel = 1.466, Opt = 1.735, and Hop = 1.876; OCB: Court = 1.198, Cons = 1.350, Sport = 1.376, Alt = 1.500, and CV = 1.542. As shown in Table 2, in general, the standard deviation values are smaller than the mean values, reflecting a good representation of the overall data. The correlation analysis results in all research variables’ indicators show significant relationships with the other variables’ indicators at the *p* < .01 level. This shows that all indicators have a mutual relationship with each other.

The measurement model estimate—by confirmatory factor analysis—is presented in Table 3. The factors’ loading values of all indicators and items equal ≥.3 (Costello and Osborne 2005), indicating validity. This means that all indicators and items—as manifested variables—can measure all research variables as latent variables. Meanwhile, reliability was determined based on the construct reliability (CR) and variance extracted (VE) values. The CR values of all variables are greater than .70, and the VE values of all variables are greater than .50, indicating a good reliability and an acceptable convergence (Hair et al. 2018).

As seen in Table 4, the goodness of fit (GOF) indices from the eleven measurements of the criterion showed that eight good indices and one marginal index were suitable; however, two others were not (chi-square and sig. probability values). According to Hair et al. (2018), the chi-square test is highly sensitive to large sample sizes (>200); it requires accompaniment by another testing method. This study included 450 teachers; therefore, the chi-square test and sig. probability values were rendered ineffective. Nevertheless, it was considered valid, since the other nine criteria tested have suitable requirements.

**Figure 1:**
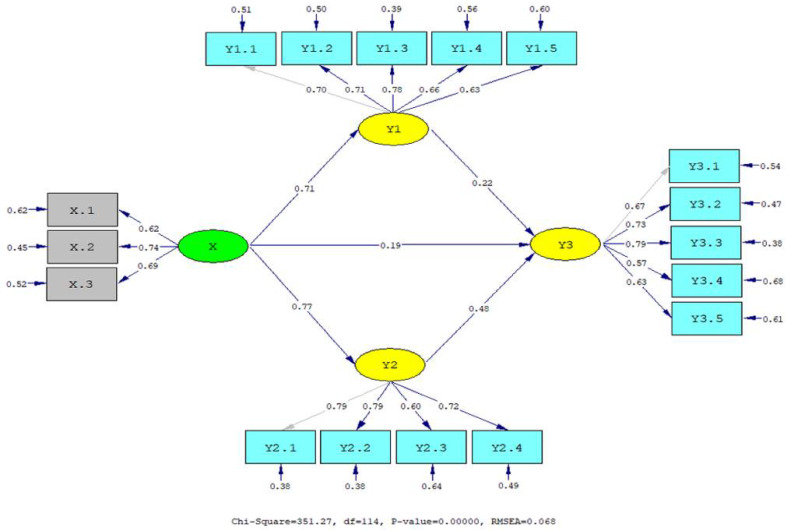
Standardized structural model.

**Figure 2:**
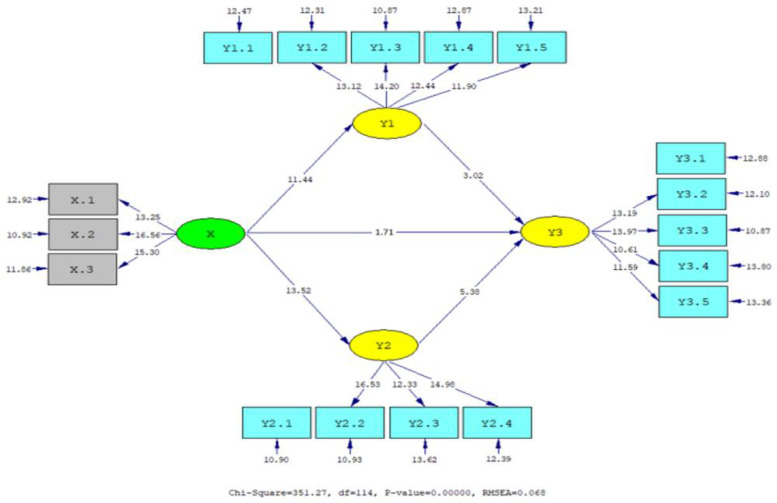
T-value structural model.

The results from the hypothesis tests are visualized in Figure 1 and Figure 2 and summarized in Table 5. All the hypotheses were supported (*t*-value > t table at α = .01 and .05). Cultural intelligence has a significant relationship with interpersonal communication (γ = .71, *p* < .01), PsyCap (γ = .77, *p* < .01), and OCB (γ = .19, *p* < .05). Interpersonal communication has a significant relationship with OCB (β = .22, *p* < .01), and PsyCap has a significant relationship with OCB (β = .48, *p* < .01). The path coefficient of the direct relationship between cultural intelligence and interpersonal communication (γ = .71) and PsyCap (γ = .77) is better than that between cultural intelligence and OCB (γ = .19). Meanwhile, interpersonal communication and PsyCap have a significant relationship with OCB. This opens up opportunities for the indirect relationship between cultural intelligence and OCB, mediated by interpersonal communication and PsyCap. Therefore, it is important to analyze the indirect relationship between cultural intelligence and OCB, mediated by interpersonal communication and PsyCap.

In Table 6, the indirect relationship between cultural intelligence and OCB, mediated by interpersonal communication and PsyCap, was found to be significant. Cultural intelligence has a significant relationship with OCB mediated by interpersonal communication (β = .16, *p* < .01) and PsyCap (β = .37, *p* < .01). The path coefficient of the indirect relationship between cultural intelligence and OCB mediated by PsyCap (.37) is better than the direct relationship between cultural intelligence and OCB (.19). This indicates the vital role of PsyCap in mediating the influence of cultural intelligence on OCB, along with interpersonal communication, which also significantly mediates the influence of cultural intelligence on OCB.

Similar results were also obtained when simulating with alternative models by eliminating mediation. The relationship between cultural intelligence, interpersonal communication, and PsyCap with OCB was significant, with indicated path coefficients of .20, .21, and .49, respectively. Furthermore, the influence of cultural intelligence and PsyCap experienced a slight increase (.19 to .20; 48 to 49), while interpersonal communication experienced a slight decrease (.22 to .21). This indicates that cultural intelligence significantly affects OCB with or without mediation. However, with PsyCap mediation, the relationship was more robust (.19 to .37). Meanwhile, with interpersonal communication mediation, the relationship was slightly weaker (.19 to .16), but with a greater degree of significance (.05 to .01). Overall, with mediation, the relationship tended to be stronger, indicating that the new empirical model of the influence of cultural intelligence on OCB with the mediation of interpersonal communication and PsyCap is rational and applicable.

## 5. Discussion

This research found that cultural intelligence significantly affects teachers’ interpersonal communication, PsyCap, and OCB. This finding confirms that cultural intelligence is a crucial determinant for teachers’ interpersonal communication, PsyCap, and OCB. This empirical result shows that teachers with high cultural intelligence tend to have adequate interpersonal communication skills; in other words, cultural intelligence can improve interpersonal communication skills. This empirical result aligns with and confirms previous studies that suggest that cultural intelligence affects interpersonal communication (Bahrami and Narafshan 2018; Henderson et al. 2018; Bostan et al. 2021). In reality, teachers who have knowledge regarding culture, including what culture is, how cultures are different, and how culture influences behavior and skills, are open-minded and use the context of a situation to support their understanding and demonstrate appropriate behaviors or social skills in new cultural settings (Thomas and Inkson 2017). Moreover, they tend to be open, empathetic, supportive and positive and to understand equality (DeVito 2016) in fostering communication with other people (including students) from various cultural backgrounds in the school environment. For teachers, interpersonal communication skills are vital. Interpersonal communication is necessary for building interactions and social relations with school members, especially students—individually and in group (classical) settings. In the modern, predominantly student-centered learning environment, that relies on a participatory and collaborative approach, interpersonal communication is a key factor that determines the continuity of learning activities. Teachers’ skills in managing a classroom are dynamic, allowing students to collaborate and participate well and effectively in learning activities; in practice, interpersonal communication skills are necessary. This study shows that these interpersonal communication skills can be achieved through improvements in cultural intelligence.

This study also indicates that cultural intelligence significantly influences PsyCap. This empirical result shows that teachers with high cultural intelligence tend to possess adequate PsyCap; in other words, cultural intelligence can be relied upon to build an improved PsyCap. This finding is consistent with previous studies conducted by Chen and Chen (2018), Mohammadi et al. (2020), and Jiony et al. (2021), in which cultural intelligence had a significant relationship with PsyCap. In practice, teachers who have the knowledge, open-mindedness, and demonstrate appropriate behaviors towards cultures across various contexts (Thomas and Inkson 2017) tend to possess adequate PsyCap, which manifests as self-efficacy, optimism, hope, and resilience (Luthans and Youssef-Morgan 2017). For teachers, PsyCaps such as self-efficacy, optimism, hope, and resilience are essential. For instance, self-efficacy—an individual’s belief in his or her capabilities to complete tasks—is necessary for teachers to build confidence in themselves in order to carry out teaching tasks well and effectively. In addition, optimism is the clear appraisal and judgment of current situations and understanding what can be done in such situations; teachers also need to build optimism when carrying out teaching tasks. Likewise, hope is an individual’s belief in his or her determination to achieve his or her goals and find possible pathways to overcome difficulties. Importantly, teachers must build more hope in order to deliver successful teaching tasks. Finally, resilience, the ability to recover from adversity or setbacks, proactively rise to challenges, and adapt to ever-changing organizational environments, is an important asset for teachers, so that they can face various difficulties and respond enthusiastically and boldly to future challenges and opportunities. Once again, this study demonstrates the power of cultural intelligence to build an improved PsyCap.

This study also showed that cultural intelligence has a significant influence on OCB. This empirical result suggests that teachers with high cultural intelligence tend to also have high OCB. In other words, cultural intelligence can increase OCB. This finding is consistent with the studies of Narayanan and Nirmala (2016), Shafieihassanabadi and Pourrashidi (2019), Mehdipour et al. (2019), and Kadam et al. (2021), who claimed that cultural intelligence has a significant relationship with OCB. Regarding the dynamics of teacher activities in schools, teachers who possess knowledge and open-mindedness and can demonstrate appropriate behaviors towards cultures in various contexts (Thomas and Inkson 2017) tend to assimilate with school members from different cultural backgrounds. Therefore, they possess strong altruism, conscientiousness, sportsmanship, courtesy, and civic virtue (Organ et al. 2006). For example, teachers with high knowledge about culture, including what culture is, how cultures are different, and how culture influences behavior and skills, tend to have good conscientiousness, courtesy, and civic virtue. Moreover, if teachers are open-minded and use the context of a situation to support their understanding and demonstrate appropriate behaviors or social skills in new cultural settings, they can easily build altruism and sportsmanship. For teachers, OCB is crucial. OCB indicators, such as altruism, conscientiousness, sportsmanship, courtesy, and civic virtue, are indispensable for teachers to deal with various problems that standardized conventional procedures and methods cannot resolve. For example, students who have difficulty following class lessons require additional assistance from teachers outside the classroom. Students who are not motivated to learn—for various reasons—should also seek assistance from a teacher. Likewise, students who behave defiantly, e.g., bullying, truancy, and brawls, also require the teacher’s input to overcome these problems. In summary, teacher OCB is not only limited to learning activities but also involves various activities and problems outside of school learning that require participation and contribution to solving problems, such as helping students who behave defiantly. At the organizational level, teacher OCB is needed by schools to help deal with various school problems that have not been resolved or to support school efforts to achieve improved progress and competitiveness. Previous studies have shown that OCB—at the individual level—can increase productivity (Barsulai et al. 2019) and performance (Hidayah and Harnoto 2018), and—at the organizational level—can help improve organizational performance (Aval et al. 2017). This means that cultural intelligence has not only a direct positive impact on teacher OCB but also indirect implications for school performance.

In addition, this study also reveals that interpersonal communication is significantly related to OCB. This evidence shows that teachers with adequate interpersonal communication skills tend to have strong OCB. In other words, interpersonal communication can improve teachers’ OCB. This finding agrees with previous studies conducted by Ezerman and Sintaasih (2018), Putra (2018), Herfina and Wulandari (2019), Nofia et al. (2019), and (Syamsudin and Retnowati 2019), in which interpersonal communication was found to be related to OCB. However, interpersonal communication, which manifests as openness, empathy, supportiveness, positiveness, and equality (DeVito 2016), is important or a predisposition that allows teachers to demonstrate strong altruism, conscientiousness, sportsmanship, courtesy, and civic virtue (Organ et al. 2006). For example, teachers who uphold the values of openness, empathy, and equality in communicating will tend to show strong altruism, courtesy, and civic virtue. Likewise, teachers with high supportiveness and positiveness tend to show strong conscientiousness and sportsmanship.

This study also indicated that PsyCap is significantly related to OCB, confirming that teachers with adequate PsyCap tend to have strong OCB. This means that Psycap can be a vital asset for teachers to develop their OCB. These findings confirm scholarly studies in which PsyCap was found to be related to OCB (Yildiz 2019; Chamisa et al. 2020; Rodríguez-Cifuentes et al. 2020; Da et al. 2021). In reality, PsyCap indicators, such as self-efficacy, optimism, hope, and resilience (Luthans and Youssef-Morgan 2017), are essential antecedents for teachers to build altruism, conscientiousness, sportsmanship, courtesy, and civic virtue (Organ et al. 2006). As an example, teachers with high efficacy and optimism tend to show strong sportsmanship. Likewise, teachers possessing high hope and resilience also tend to show strong conscientiousness and civic virtue; therefore, it is easy for them to help other parties, such as students and schools, who may require assistance.

In addition, this study also discovered new empirical data on the role of interpersonal communication and PsyCap in mediating the relationship between cultural intelligence and teachers’ OCB. Both interpersonal communication and PsyCap significantly mediated the relationship between cultural intelligence and teachers’ OCB. However, PsyCap’s mediating role was more enhanced than interpersonal communication, which means that PsyCap is more dominant and crucial in mediating the relationship between cultural intelligence and teachers’ OCB compared to interpersonal communication. Moreover, these findings led to a new empirical model regarding the relationship between cultural intelligence and OCB mediated by interpersonal communication and PsyCap. In contrast, the results were also significant when simulating with alternative models by eliminating mediation. The relationship between cultural intelligence and PsyCap experienced a slight increase, while interpersonal communication experienced a slight decrease. Overall, with mediation, the relationship tended to be stronger, indicating that the new empirical model of the relationship between cultural intelligence and OCB mediated by interpersonal communication and PsyCap is rational and applicable. Naturally, this finding can be used as a topic for discussion among researchers and practitioners. Furthermore, it can also be adopted as a model for developing cultural intelligence, especially for improving interpersonal communication, PsyCap, and OCB across various locations, sectors, organizations, and contexts.

Overall, the results of this study indicate that the strength of cultural intelligence relates to interpersonal communication, PsyCap, and teacher OCB. Therefore, the cultural intelligence of teachers should be continuously improved by using an appropriate strategy. First, teachers should independently and consciously increase their cultural intelligence capacity by reading literature relevant to cultural intelligence. Second, principals should encourage teachers to participate in training programs specifically designed to improve teachers’ cultural intelligence. These training programs should involve instructors from expert circles who are competent in cultural intelligence. Importantly, the provided training material should lead to the mastery of knowledge regarding cultures, including: (1) what culture is, how cultures are different, and how culture influences behavior and skills; (2) to be open-minded and use the context of a situation to support understanding; and (3) to demonstrate appropriate behaviors or social skills in new cultural settings. Moreover, the methods should be used according to the needs of the training material, i.e., combination: discussions—including focus group discussions, simulations, and role-playing. Lastly, school principals should create and enforce guidelines for teachers to think and behave culturally in the school environment.

## 6. Conclusions

Cultural intelligence is an individual’s ability to recognize, understand, and adapt to cross-cultural contexts to change his or her self-capacity. Therefore, cultural intelligence contributes significantly to the lives of individuals, groups, and organizations. This research found that cultural intelligence significantly affects interpersonal communication, PsyCap, and OCB among teachers in Indonesia. Furthermore, this study also produced a new model regarding the relationship between cultural intelligence and teachers’ OCB mediated by interpersonal communication and PsyCap. This finding is crucial for teachers’ well-being, especially in developing teachers’ intercultural competence, including work-related outcomes, such as job satisfaction, work adjustment, knowledge sharing, and job performance, which can impact the team and organizational performance. Therefore, in the future, researchers and practitioners should discuss and adopt a new empirical model to increase cultural intelligence, in order to specifically enhance teachers’ interpersonal communication, PsyCap, and OCB, in various contexts.

## 7. Limitations and Future Research

The findings of this study should be interpreted by considering its limitations. The mediating effects produced by this study should be interpreted with caution in relation to the cross-sectional study design. Accordingly, future research should investigate the interrelationships between cultural intelligence, interpersonal communication, and psychological capital using a longitudinal or cross-lagged panel design to obtain stronger conclusions about the causal order of these variables. Besides, this study could not control all variables that may have interfered with the relationship between cultural intelligence, PsyCap, interpersonal communication, and OCB, e.g., the Big Five personality traits. Future research may involve the Big Five personality traits, both as an antecedent of cultural intelligence and a means of moderating the influence of cultural intelligence on interpersonal communication, PsyCap, and OCB. Moreover, this study did not accommodate all indicators/dimensions of all the research variables. Further research should utilize indicators/dimensions not used in this study or comprehensively synthesize all indicators/dimensions. Furthermore, this study did not explore the empirical facts of why cultural intelligence affects OCB directly or indirectly—mediated by interpersonal communication and PsyCap. Therefore, further research should respond to these limitations using mixed methods—simultaneously, using both quantitative and qualitative analyses. Finally, further research should replicate the findings of this research by adding different data sources (participants) for OCB, such as principals and/or students.

## Figures and Tables

**Table 1:** Profile of the research participants.

Profile	Amount	Percentage
Gender		
Male	141	31.33
Female	309	68.67
Age		
≤25 years	34	7.56
26–35 years	159	35.33
36–45 years	111	24.67
46–55 years	111	24.67
≥56 years	35	7.78
Education		
Diploma (D3)	19	4.22
Bachelor (S1)	405	90
Postgraduate (S2)	26	5.78
Doctoral (S3)	0	0
Status		
Married	360	80
Unmarried	90	20
Experience		
≤5 years	127	28.22
6–10 years	83	18.44
11–15 years	100	22.22
≥16 years	140	31.11

**Table 2:** Descriptive statistics and correlation matrices.

Variables	Mean	Std. Dev.	1	2	3	4	5	6	7	8	9	10	11	12	13	14	15	16	17
**Cultural Intelligence**																			
Know	8.85	1.127	1.00																
Mind	9.04	0.925	.51 **	1.00															
BS	8.46	1.107	.41 **	.52 **	1.00														
**Interpersonal Communication**																			
Open	9.09	1.020	.40 **	.38 **	.36 **	1.00													
Emp	8.40	1.261	.27 **	.31 **	.36 **	.51 **	1.00												
Sup	8.72	1.140	.30 **	.37 **	.39 **	.56 **	.54 **	1.00											
Pos	4.18	0.733	.25 **	.28 **	.26 **	.43 **	.49 **	.52 **	1.00										
Equ	4.23	0.744	.22 **	.36 **	.39 **	.38 **	.47 **	.49 **	.45 **	1.00									
**PsyCap**																			
Sel	12.56	1.466	.37 **	.48 **	.40 **	.36 **	.31 **	.36 **	.41 **	.32 **	1.00								
Opt	11.74	1.735	.32 **	.34 **	.39 **	.29 **	.26 **	.28 **	.33 **	.28 **	.64 **	1.00							
Hop	11.09	1.876	.19 **	.19 **	.32 **	.21 **	.21 **	.23 **	.26 **	.21 **	.42 **	.58 **	1.00						
Res	13.02	1.383	.40 **	.54 **	.46 **	.41 **	.30 **	.34 **	.31 **	.31 **	.55 **	.53 **	.41 **	1.00					
**OCB**																			
Alt	7.98	1.500	.21 **	.29 **	.29 **	.28 **	.22 **	.35 **	.36 **	.25 **	.42 **	.43 **	.34 **	.34 **	1.00				
Cons	7.97	1.350	.28 **	.36 **	.31 **	.26 **	.26 **	.36 **	.31 **	.28 **	.46 **	.44 **	.34 **	.36 **	.51 **	1.00			
Sport	7.82	1.376	.34 **	.37 **	.38 **	.33 **	.31 **	.40 **	.34 **	.33 **	.43 **	.38 **	.37 **	.40 **	.56 **	.63 **	1.00		
16. Court	8.90	1.198	.35 **	.44 **	.37 **	.31 **	.24 **	.30 **	.24 **	.21 **	.37 **	.33 **	.17 **	.40 **	.36 **	.39 **	.41 **	1.00	
17. CV	7.99	1.542	.36 **	.40 **	.38 **	.34 **	.33 **	.35 **	.30 **	.28 **	.43 **	.41 **	.30 **	.44 **	.39 **	.39 **	.48 **	.40 **	1.00

** *p* < .01.

**Table 3:** Results of the measurement model.

Variables	Indicators	Items	Factor Loading		CR	VE
			Item	Indicator		
Cultural intelligence	Know	I understand the meaning of culture and its diversity	.82	.69	.880	.558
		I understand how culture affects behavior	.82			
	Mind	I respect the opinions of other people from different cultural backgrounds	.79	.95		
		I learn from the lives of other people from different cultural backgrounds.	.76			
	BS	I easily interact socially with people from different cultural backgrounds	.78	.89		
		I quickly adjust to a new culture	.44			
Interpersonal communication	Open	I provide information honestly (according to the facts) to others	.57	1.00	.838	.523
		I welcome input from other people	.72			
	Emp	I am enthusiastic about listening to other people when they talk	.71	.89		
		I trust other people when their share experiences	.59			
	Sup	I respect the uniqueness of the interlocutor	.71	1.03		
		I show support for the other person’s opposing views	.53			
	Pos	I sincerely appreciate the aspirations of others	.62	.84		
	Equ	I view differences as a gift of life that deserves to be cherished	.55	.77		
PsyCap	Sel	I feel able to carry out teaching tasks to the fullest	.76	.81	.894	.580
		I feel I can contribute to the progress of the school	.80			
		I feel I easily adapt to the new challenging tasks at school	.69			
	Opt	I believe in quickly providing alternative ideas for solving unresolved school problems	.68	1.01		
		I see myself as having the potential to be successful at school	.61			
		I have a strong belief in my ability to solve various future problems	.75			
	Hop	I believe in achieving personal goals as a teacher	.65	.87		
		I feel I easily complete routine tasks at school	.45			
		I am sure I am able to cope with new things at school	.63			
	Res	I believe in being able to overcome the difficulties of teaching assignments	.71	.74		
		I can get through difficult times at school related to educational assignments	.85			
		I am optimistic I can adapt to the demands of future teaching assignments	.75			
OCB	Alt	I actively share knowledge with other teachers even if not asked	.76	.79	.892	.572
		I help solve new problems that arise at school	.79			
	Cons	I use my work time as efficiently as possible	.47	1.12		
		I finish work earlier than required by school standards	.59			
	Sport	I accept the shortcomings in school as a challenge that needs to be fixed	.47	1.24		
		I am trying hard to solve unfinished school problems	.59			
	Court	I actively build social relations with other teachers who have different views	.81	.59		
		I try to give in to others to avoid conflict	.77			
	CV	I am active in various additional activities at school	.78	.70		
		I prioritize school interests over personal matters	.65			

**Table 4:** Goodness-of-fit statistics.

Goodness of Fit Index	Cut of Value	Result	Information
Absolute fit measures			
Chi-square	χ2 < χ2 table	311.77	Poor
Sig. Probability	*p* > .05	.00	Poor
GFI	≥.09	.92	Good
RMSEA	≤.08	.068	Good
Incremental fit measures			
NFI	>.90	.96	Good
NNFI	≥.90	.97	Good
AGFI	≥.90	.89	Marginal
CFI	≥.90	.98	Good
RFI	≥.90	.96	Good
Parsimony fit measures			
Normed chi-square	1–2 or < 3	1.08	Good
PNFI	0–1	.81	Good

**Table 5:** Hypothesis testing results.

Hypothesis	β/γ	T-Value	Decision
H1. Cultural intelligence (X) and interpersonal communication (Y_1_)	.71 **	11.44	Supported
H2. Cultural intelligence (X) and PsyCap (Y_2_)	.77 **	13.52	Supported
H3. Cultural intelligence (X) and OCB (Y_3_)	.19 *	1.71	Supported
H4. Interpersonal communication (Y_1_) and OCB (Y_3_)	.22 **	3.02	Supported
H5. PsyCap (Y_2_) and OCB (Y_3_)	.48 **	5.38	Supported

* *p* < .05; ** *p* < .01.

**Table 6:** Mediation relationship analysis.

Indirect Relationship	β	Z-Value	Decision
Cultural intelligence (X) and OCB (Y_3_) mediated by interpersonal communication (Y_1_)	.16 **	9.28	Supported
Cultural intelligence (X) and OCB (Y_3_) mediated by PsyCap (Y_2_)	.37 **	10.79	Supported

** *p* < .01.

## Data Availability

Not applicable.

## References

[B1-jintelligence-10-00003] Abu-Bader Soleman, Jones Tiffanie Victoria (2021). Statistical mediation analysis using the Sobel Test and hayes SPSS process macro. International Journal of Quantitative and Qualitative Research Methods.

[B2-jintelligence-10-00003] Aderibigbe John K., Mjoli Tembha Q. (2018). Evaluation of the components of psychological capital and organizational citizenship behavior among Nigerian graduate employees. Journal of Economics and Behavioral Studies.

[B3-jintelligence-10-00003] Adillah Mirrah Nur, Sidin Indahwaty, Amqam Hasnawati (2019). Impact of psychological capital on organizational citizenship behavior (OCB) towards male and female nurses in teaching hospital. Dama Academic Scholarly Journal of Researchers.

[B4-jintelligence-10-00003] Ahmadian Elham, Amirpour Mahnaz (2018). The effect of cultural intelligence on communication skills. Journal of Fundamentals of Mental Health.

[B5-jintelligence-10-00003] Akhal Khalid, Liu Shimin (2019). Cultural intelligence effects on expatriates’ adjustment and turnover intentions in Mainland China. Management Research Review.

[B6-jintelligence-10-00003] Ang Soon, Van Dyne Lin, Ang Soon, Van Dyne Linn (2008). Conceptualization of cultural intelligence: Definition, distinctiveness, and nomological network. Handbook of Cultural Intelligence: Theory, Measurement, and Applications.

[B7-jintelligence-10-00003] Ang Soon, Ng Kok Yee, Rockstuhl Thomas, Sternberg Robert J. (2020). Cultural intelligence. Cambridge Handbook of Intelligence.

[B8-jintelligence-10-00003] Ang Soon, Rockstuhl Thomas, Tan Mei Ling (2015). Cultural intelligence and competencies. International Encyclopedia of the Social & Behavioral Sciences.

[B9-jintelligence-10-00003] Aval Saleh Moradi, Haddadi Ebrahim, Keikha Aleme (2017). Investigating the impact of organizational citizenship behavior (OCB) components on organizational agility. Interdisciplinary Journal of Education.

[B10-jintelligence-10-00003] Bahrami Anahid, Narafshan Mehry Haddad (2018). Enhancing cultural intelligence and interpersonal communication: Multi-authenticity exposure in English as a foreign language (EFL) context. Journal of Education and Culture Studies.

[B11-jintelligence-10-00003] Baluku Martin Mabunda, Mugabi Eriphase Nsaale, Nansamba Joyce, Matagi Leonsio, Onderi Peter, Otto Kathleen (2021). Psychological capital and career outcomes among final year university students: The mediating role of career engagement and perceived employability. International Journal of Applied Positive Psychology.

[B12-jintelligence-10-00003] Baluku Martin Mabunda, Kikooma Julius Fred, Bantu Edward, Onderi Peter, Otto Kathleen (2019). Impact of personal cultural orientations and cultural intelligence on subjective success in selfemployment in multi-ethnic societies. Journal of Global Entrepreneurship Research.

[B13-jintelligence-10-00003] Barsulai Stella C., Makopondo Richard O. B., Fwaya Erick V. O. (2019). The impact of organizational citizenship behavior on employee productivity in star-rated hotels in Kenya. European Journal of Hospitality and Tourism Research.

[B14-jintelligence-10-00003] Berraies Sarra (2020). Effect of middle managers’ cultural intelligence on firms’ innovation performance. Personnel Review.

[B15-jintelligence-10-00003] Bostan Zahra, Honari Habib, Karoubi Mehdi, Nikjoo Adel (2021). The relationship between cultural intelligence (CQ), spiritual intelligence (SQ) and interpersonal communication skills: The case of cultural tour guides. Tourism Culture & Communication.

[B16-jintelligence-10-00003] Carmona-Halty Marcos, Schaufeli Wilmar B., Salanova Marisa (2019). Good relationships, good performance: The mediating role of psychological capital-a three-wave study among students. Frontiers in Psychology.

[B17-jintelligence-10-00003] Cascio Wayne F. (2016). Managing Human Resources: Productivity, Quality of Work-Life, Profit.

[B18-jintelligence-10-00003] Cavus Mustafa Fedai, Gokcen Ayse (2015). Psychological capital: Definition, components, and effects. British Journal of Education, Society &Behavioural Science.

[B19-jintelligence-10-00003] Chamisa Shingirayi F., Mjoli Temba Q., Mhlanga Tatenda S. (2020). Psychological capital and organizational citizenship behavior in selected public hospitals in the Eastern Cape Province of South Africa. SA Journal of Human Resource Management/SATydskrifvirMenslikehulpbronbestuur.

[B20-jintelligence-10-00003] Chen Angela Shin-Yih, Chen Wei-Tung (2018). The relationship between cultural intelligence and job involvement with the mediating effect of PsyCap. Paper presented at Seventeenth Asia-Pacific Conference on Global Business, Economics, Finance & Social Sciences (AP18 Hong Kong Conference).

[B21-jintelligence-10-00003] Costello Anna B., Osborne Jason (2005). Best practices in exploratory factor analysis: Four recommendations for getting the most from your analysis. Practical Assessment, Research, and Evaluation.

[B22-jintelligence-10-00003] Cronk Lee (2017). Culture’s influence on behavior: Steps toward a theory. Evolutionary Behavioral Sciences.

[B23-jintelligence-10-00003] Da Shu, Zhu Ze, Cen Hongyu, Gong Xianmin, Siu Oi Ling, Zhang Xichao (2021). Psychological capital, positive affect, and organizational outcomes: A three-wave cross-lagged study. Journal of Pacific Rim Psychology.

[B24-jintelligence-10-00003] DeVito Joseph A. (2016). The Interpersonal Communication Book.

[B25-jintelligence-10-00003] Duong Hue Trong, Nguyen Long Thang Van, McFarlane Soroya Julian, Nguyen HoaThanh, Nguyen Khai The (2021). Preventing the COVID-19 Outbreak in Vietnam: Social Media Campaign Exposure and the Role of Interpersonal Communication. Health Communication.

[B26-jintelligence-10-00003] Earley P. Christopher, Ang Soon (2003). Cultural intelligence: Individual Interactions Across Cultures.

[B27-jintelligence-10-00003] El-Zohiry Amira A., Abd-Elbaqy Khadeeja Y. (2019). The moderating effect of intrinsic motivation on the relationship between psychological capital and organizational citizenship behaviors. Management Review: An International Journal.

[B28-jintelligence-10-00003] Ezerman Maria Marina, Sintaasih Desak Ketut (2018). Effect of servant leadership, trust in leadership on organizational citizenship behavior with interpersonal communications as mediation variables. IOSR Journal of Business and Management (IOSR-JBM).

[B29-jintelligence-10-00003] Gibson James L., Ivancevich Jhon M., Donnely James D., Konopaske Robert (2012). Organizations: Behavior, Structure, and Processes.

[B30-jintelligence-10-00003] Goertzen Brent, Whitaker Brett L. (2015). Development of psychological capital in an academic-based leadership education program. Journal of Management Development.

[B31-jintelligence-10-00003] Hair Joseph F., Babin Barry J., Anderson Rolph E., Black William C. (2018). Multivariate Data Analysis.

[B32-jintelligence-10-00003] Hellriegel Don, Slocum Jhon W. (2011). Organizational Behavior.

[B33-jintelligence-10-00003] Henderson Linda S., Stackman Richard W., Lindekilde Rikke (2018). Why cultural intelligence matters on global project teams. International Journal of Project Management.

[B34-jintelligence-10-00003] Herfina, Wulandari Dian (2019). Improving the organizational citizenship behavior (OCB) through developing effective interpersonal communication and transformational leadership. JHSS Journal of Humanities and Social Studies.

[B35-jintelligence-10-00003] Hidayah Siti, Harnoto Harnoto (2018). Role of organizational citizenship behavior (OCB), perception of justice, and job satisfaction on employee performance. Jurnal Dinamika Manajemen.

[B36-jintelligence-10-00003] Hitt Michael A., Miller C. Chet, Colella Adrienne (2011). Organizational Behavior.

[B37-jintelligence-10-00003] Imran Mohammad, Shahnawaz Mohammad Ghazi (2020). PsyCap and performance: Well-being at work as a mediator. Asia-Pacific Journal of Management Research and Innovation.

[B38-jintelligence-10-00003] Jiony Mary Monica, Lew Tek Yew, Gom Daria, Tanakinjal Geoffrey Harvey, Sondoh Stephen (2021). Influence of cultural intelligence and psychological capital on service quality: A study of the hotel industry in Sabah, Malaysia. Sustainability.

[B39-jintelligence-10-00003] Kadam Raavee, Balasubramanian Sreejith, Abdul Waheed Kareen, Jebeen Shazi S. (2021). Predicting organizational citizenship behavior in a multicultural environment: The role of cultural intelligence and cultural distance. International Journal of Cross Cultural Management.

[B40-jintelligence-10-00003] Kastanakis Minas N., Voyer Benjamin G. (2014). The effect of culture on perception and cognition: A conceptual framework. Journal of Business Research.

[B41-jintelligence-10-00003] Kong Fanzhu, Tsai Cheng-Hung, Tsai Fu-Sheng, Huang Wenti, Cruz Shareena Malapitan de la (2018). Psychological capital research: A meta-analysis and implications for management sustainability. Sustainability.

[B42-jintelligence-10-00003] Kreitner Robert, Kinicki Angelo (2013). Organizational Behavior.

[B43-jintelligence-10-00003] Leung Kwok, Ang Soon, Tan Mei Ling (2014). Intercultural competence. Annual Review of Organizational Psychology and Organizational Behavior.

[B44-jintelligence-10-00003] Luthans Fred, Youssef Carolyn M. (2004). Human, social, and now positive psychological capital management: Investing in people for competitive advantage. Organizational Dynamics.

[B45-jintelligence-10-00003] Luthans Fred, Youssef-Morgan Carolyn M. (2017). Psychological capital: An evidence-based positive approach. Reviews in Advance.

[B46-jintelligence-10-00003] Matsumoto David, Hwang Hyisung C. (2013). Assessing cross-cultural competence: A review of available tests. Journal of Cross-Cultural Psychology.

[B47-jintelligence-10-00003] McShane Steven L., Von Glinow Mary A. (2020). Organizational Behavior: Emerging Knowledge, Global Reality.

[B48-jintelligence-10-00003] Mehdipour Yousef, Rashki Toba, Rashki Fatemeh (2019). Cultural intelligence, organizational justice and citizenship behavior in women teachers (Educational Management Project). Journal of Organizational Behavior Research.

[B49-jintelligence-10-00003] Mohammadi Sara, Nadaf Mahdi, Roshan Sara (2020). The impact of emotional intelligence and cultural intelligence on resistance to changing employees with the mediating role of psychological capital. Quarterly Social Psychology Research.

[B50-jintelligence-10-00003] Muhammad Khadijatu, Toryila Asombo Shadrach, Saanyol Daniel Benjamin (2018). The role of interpersonal relationship on job performance among employees of Gboko Local Government Area of Benue State, Nigeria. International Journal of Social Sciences and Management Research.

[B51-jintelligence-10-00003] Mukherji Shoma, Jainb Neera, Sharmab Radha R. (2016). Relevance of cultural intelligence and communication effectiveness for global leadership preparedness: Study of Indian managers. Journal of International Business Research and Marketing.

[B52-jintelligence-10-00003] Mukhtar Risnita, Prasetyo Muhammad A. M. (2020). The influence of transformational leadership, interpersonal communication, and organizational conflict on organizational effectiveness. International Journal of Educational Review.

[B53-jintelligence-10-00003] Narayanan Balasubramanian Lalkshmi, Nirmala D. (2016). A study of cultural intelligence of employees and its relationship with organizational citizenship behavior in a multinational company. IOSR Journal of Humanities and Social Science (IOSR-JHSS).

[B54-jintelligence-10-00003] Nawaz Muhammad, Abid Ghulam (2019). Does prosocial motivation and psychological capital improve organizational citizenship behavior? An emperical study through the moderating role of workplace incivility. Journal Research Square.

[B55-jintelligence-10-00003] Nofia Desi, Yasri, Abror (2019). The effects of interpersonal communication and organizational commitment on organizational citizenship behavior (at Agam District Government). Advances in Economics, Business and Management Research.

[B56-jintelligence-10-00003] Organ Dennis W., Podsakoff Philip M., MacKenzie Scott B. (2006). Organizational Citizenship Behavior: Its Nature, Antecedents, and Consequences.

[B57-jintelligence-10-00003] Popescu R. Nicoleta, Fistung Daniel F., Popescu Teodor, Popescu Alexandra M. (2018). Is the organizational citizenship behavior (OCB) a predictor for the cultural intelligence (CQ)?. Procedia—Social and Behavioral Sciences.

[B58-jintelligence-10-00003] Putra Ramdani Bayu (2018). Effect of organization commitment mediation to organizational citizenship behaviors with interpersonal communication and work satisfaction as antecedents variable. E-Jurnal Apresiasi Ekonomi.

[B59-jintelligence-10-00003] Rockstuhl Tohmas, Van Dyne Lin (2018). A bi-factor theory of the four-factor model of cultural intelligence: Meta-analysis and theoretical extensions. Organizational Behavior and Human Decision Processes.

[B60-jintelligence-10-00003] Rodríguez-Cifuentes Francisco, Segura-Camacho Adrian, García-Ael Cristina, Topa Gabriela (2020). The mediating role of psychological capital between motivational orientations and their organizational consequences. International Journal of Environmental Research and Public Health.

[B61-jintelligence-10-00003] Saraih Ummi Naiemah, Azmi A. H., Sakdan Mohd Fo, Mohd Karim K., Amlus M. Harith (2019). Understanding the effects of interpersonal communication and task design on job performance among employees in the manufacturing company. Humanities & Social Sciences Reviews.

[B62-jintelligence-10-00003] Schlaegel Christopher, Richter Nicole F., Taras Vasyl (2021). Cultural intelligence and work-related outcomes: A meta-analytic examination of joint effects and incremental predictive validity. Journal of World Business.

[B63-jintelligence-10-00003] Schultz Duane, Schultz Sydney E. (2016). Psychology and Work Today.

[B64-jintelligence-10-00003] Shafieihassanabadi Sobhan, Pourrashidi Rostam (2019). An analysis of emotional and cultural intelligence relationship on organizational citizenship behaviors among employees of Iranian tourism organizations. International Transaction Journal of Engineering, Management, & Applied Sciences & Technologies.

[B65-jintelligence-10-00003] Slatten Terje, Mutonyi Barbara R., Lien Gudbrand (2020). The impact of individual creativity, psychological capital, and leadership autonomy support on hospital employees’ innovative behavior. BMC Health Services Research.

[B66-jintelligence-10-00003] Solomon A., Steyn Renier (2017). Cultural intelligence: Concepts and definition statements. South African Journal of Business Management.

[B67-jintelligence-10-00003] Sternberg Robert J., Wong Chak H., Kreisel Anastasia P. (2021). Understanding and assessing cultural intelligence: Maximum-performance and typical-performance approaches. Journal of Intelligence.

[B68-jintelligence-10-00003] Stoermer Sebastian, Davies Samuel, Froese Fabian J. (2021). The influence of expatriate cultural intelligence on organizational embeddedness and knowledge sharing: The moderating effects of host country context. Journal of International Business Studies.

[B69-jintelligence-10-00003] Syamsudin Billy Tunas, Retnowati Rita (2019). Enhancing organizational citizenship behavior (OCB) through transformational leadership, interpersonal communication, and work motivation. International Journal of Managerial Studies and Research (IJMSR).

[B70-jintelligence-10-00003] Tang Yuan, Shao Yun-Fei, Chen Yu-Jun (2019). Assessing the mediation mechanism of job satisfaction and organizational commitment on innovative behavior: The perspective of psychological capital. Frontier Psychology.

[B71-jintelligence-10-00003] Thomas David C., Inkson Kerr (2017). Cultural Intelligence: Living and Working Globally.

[B72-jintelligence-10-00003] Van Griethuijsen Ralf A. L. F., van Eijck Michiel W., Haste Helen, Den Brok Perry J., Skinner Nigel C., Mansour Nasser, Gencer Ayse S., BouJaoude Saouma (2015). Global patterns in students’ views of science and interest in science. Research in Science Education.

[B73-jintelligence-10-00003] Wang Yanfei, Chen Yi, Zhu Yu (2021). Promoting innovative behavior in employees: The mechanism of leader psychological capital. Frontiers in Psychology.

[B74-jintelligence-10-00003] Widodo Widodo (2019). Metodologi Penelitian Populer & Praktis [Popular & Practical Research Methodologies].

[B75-jintelligence-10-00003] Widodo Widodo, Gustari Irvandi (2020). Teacher’s innovative behavior in Indonesian school: The role of knowledge management, creativity, and OCB. Universal Journal of Educational Research.

[B76-jintelligence-10-00003] Yari Nooria, Lankut Erik, Alon Ilan, Richter Nicole F. (2020). Cultural intelligence, global mindset, and crosscultural competencies: A systematic review using bibliometric methods. European J. International Management.

[B77-jintelligence-10-00003] Yildiz Harun (2019). The interactive effect of positive psychological capital and organizational trust on organizational citizenship behavior. SAGE Open.

[B78-jintelligence-10-00003] Zhao Shuming, Liu Yan, Zhou Lulu (2020). How does a boundaryless mindset enhance expatriate job performance? The mediating role of proactive resource acquisition tactics and the moderating role of behavioral cultural intelligence. The International Journal of Human Resource Management.

